# Spectrophotometric detection of susceptibility to anti-malarial drugs

**DOI:** 10.1186/1475-2875-12-305

**Published:** 2013-08-30

**Authors:** Yulia M Serebrennikova, Janus Patel, Wilbur K Milhous, Luis H Garcia-Rubio, Debra E Huffman, Jennifer M Smith

**Affiliations:** 1 College of Public Health, University of South Florida, 13201 Bruce B. Downs Blvd., Tampa, FL 33612, USA; 2 Claro Scientific 10100 Dr, Martin Luther King Jr. St. N., St., Petersburg, FL 33716, USA

**Keywords:** Malaria, Antimalarial, Spectroscopy, Spectral interpretation

## Abstract

**Background:**

With malaria drug resistance increasing in prevalence and severity, new technologies are needed to aid and improve the accuracy and clinical relevance of laboratory or field testing for malaria drug resistance. This study presents a method based on simple and reagentless spectroscopic measurements coupled with comprehensive spectral interpretation analysis that provides valuable quantitative information on the morphological and compositional responses of *Plasmodium falciparum* and infected red blood cells (IRBCs) to anti-malarial treatment.

**Methods:**

The changes in the size, internal structure, nucleotide and haemozoin composition of the parasites as well as the morphology (size and shape) and haemoglobin composition of the IRBCs treated with dihydroartemisinin (DHA) and mefloquine (MFQ) were investigated using a spectral interpretation analysis.

**Results:**

DHA treatment reduced the sizes of the parasites and their structural organelles. The haemoglobin composition of the host IRBCs determined from spectroscopic analysis changed negligibly following DHA treatment. MFQ treated parasites grew to the same size as those from parallel non-treated cultures but lacked haemozoin. Lesser deformation of the cell shape and no haemoglobin depletion were detected for the IRBCs of MFQ treated cultures.

**Conclusions:**

The spectroscopic analysis method proved to be sensitive for recognition of the effects of anti-malarial treatment on the structure and composition of the parasites and IRBCs. The method can have significant potential for research and clinical applications such as evaluating patient specimens for drug action, drug effects or for therapeutic monitoring.

## Background

Microscopy has long been the “gold standard” as a tool for the research based characterization of malaria infected red blood cells (IRBCs) and for the detection of malaria parasites in the field [1-5]. Because this technique is constrained by substantial technical and economic hurdles such as operator training, time needed to perform a test, and labor intensiveness, there is an ongoing search for alternatives. Immunochromatography-based rapid diagnostic tests have gained widespread popularity because of ease-of-use but often suffer from high variability, poor sensitivity for mixed infections of *Plasmodium* species other than *Plasmodium falciparum*, extensive requirements for reagent quality control [2,3,6]. Techniques based on polymerase chain reaction (PCR) offer superior sensitivity, ability to detect mixed species infection and the ability to differentiate among four species of *Plasmodium* but they require considerable cost of equipment as well as storage and maintenance requirements for reagents [2,4]. Quantitative PCR has been repeatedly demonstrated to be more accurate than microscopy for the detection of malaria at very low levels of parasitaemia [7,8] and it disagrees with microscopy for parasitaemia counts at higher parasitaemia levels [8]. It is not straightforward and requires complicated analysis to assess the sensitivity of the malaria parasites to anti-malarials using immunochromatography or molecular methods [9-11] and is time-consuming when using classical *in vivo* studies [12].

UV-visible spectroscopy that explores spectral changes in the infected red blood cells is a new tool in malaria diagnostics that overcome most of the disadvantages of the current malaria diagnostics methods by being rapid, sensitive and quantitative. Spectroscopic and light scattering techniques have been a subject of continuous interest since these non-destructive measurements provide substantial information on the physical, chemical, and physiological character of cells and, therefore, can potentially detect and identify changes in cells that are indicative of diseases. For instance, flow cytometry and commercial haematology analyzers utilizing abnormalities in the multiple-angle polarized scattering plots have demonstrated potential for the detection of malaria parasites in blood with reported sensitivities of ≥ 95% in samples with >100 parasites/μl and is in compliance with WHO malaria-diagnostic guidelines [1,13].

Given that the asexual stages of the malaria parasites occur inside red blood cells (RBCs), assessment of the changes in the red blood cell (RBC) properties is a valuable approach for infection detection and characterization. This prominent component of blood has long been attractive to the spectroscopic investigations as a light scatterer with a homogeneous body and the distinct spectroscopic features of its main constituent, haemoglobin. The IRBC morphology and composition appreciably affect the spectra with the progression of the intraerythrocytic development of malaria parasites. Even though the size and shape of an IRBC at the ring stage remain the same as those of non-infected RBCs [14], the parasite with its parasitophorus vacuole occupies 5-15% of the host volume [15-17]. The parasite occupies about one third of the host IRBC by the trophozoite stage [16,18] and more than a half of the host volume through the subsequent schizont stage [15,18,19]. This growth is accompanied with the loss of shape, swelling, and formation of protrusions on the IRBC surface [18,20,21]. The parasite continuously uptakes haemoglobin from the host cytosol and converts it into haemozoin deposited into the parasite’s digestive vacuole [22-24]. Haemoglobin depletion of the host and haemozoin build up in the parasite’s vacuole progress with the parasite’s development and become the greatest at the schizont stage [22-24]. It has been demonstrated for different intraerythrocytic life stages of *P. falciparum* that the UV-visible spectroscopy can track these changes [25].

Since anti-malarial treatment leads to the morphological and/or compositional changes in the parasites, in this study it was hypothesized that anti-malarial effect can be captured by UV-visible measurements. The objectives of this study were to examine the hypothesis with the experimentally acquired data using dihydroartemisinin (DHA) and mefloquine (MFQ), to effectively obtain quantitative measurements of the morphological parameters, haemoglobin composition of the IRBCs and composition of the parasites following the treatment, and to show the sensitivity of the method for the recognition of the effects of anti-malarial treatment. To achieve the objectives, simultaneous measurements of the absorption and forward scattering in the UV-visible-NIR portion of the electromagnetic spectrum were coupled with a spectral interpretation model based on an ellipsoidal approximation to the multilayered Mie theory. The parameters such as the size, nucleotide and haemozoin composition of the parasites as well as the size, shape and haemoglobin composition of the IRBCs could serve as the determining factors of the susceptibility or resistance of the parasite.

## Methods

### Sample preparation

*In vitro* cultures of the W2 strain *P. falciparum* were grown at 4% hematocrit in RPMI media to 6-14% parasitaemia following the standard method [26]. Cultures were synchronized with both D-sorbitol and incubation temperature cycling of 17°C and 40°C to achieve a high concentration of ring stage parasites. Five independent experiments were conducted. In each experiment, cultures were prepared by either splitting a parent culture into subcultures, or growth of two or three parallel cultures. One subculture or parallel culture was set as a control (no drug exposure) and others were treated with either mefloquine (MFQ) or dihydroartemisinin (DHA). The exposure concentration of each drug was 1,500 ng/mL (7.5 μl of 1 mg/ml MFQ or DHA solution was added to a 5 ml culture). The experiments always started with all cultures being at the ring stage. The parasite extraction from IRBCs was performed by saponin lysis as previously described [14]. Briefly, 1 ml of 0.1% saponin solution in PBS was added to 0.5 ml of approximately 50% haematocrit RBC suspension. The mixture was incubated at 37°C with constant agitation for 15 minutes. Then, it was centrifuged at 13,000 rpm for 1 min and the pellet was resuspended in PBS. The procedure was repeated 3–4 times to remove the dissolved haemoglobin and the RBC membrane fragments. Visible microscopy inspection was performed to confirm the integrity of the extracted parasites.

### Spectrophotometric measurements

To measure the UV-visible-NIR spectra of IRBC cultures, 0.5 ml of each culture was centrifuged at 13,000 rpm for 30 seconds, growth media was aspired, and the cells were suspended in 1 ml of phosphate buffered saline (PBS). The procedure was repeated 3–4 times until the supernatant was clear of the growth media. The UV-visible-NIR spectra were recorded using a diode array spectrometer (Agilent 8453 Santa Clara, CA) having an acceptance angle smaller than 2° at room temperature using a 1 cm pathlength cuvette. The measurements were conducted with an acquisition time of 0.5 sec, signal to noise ratio greater than 1000, and 1 nm wavelength resolution. Prior to recording a sample spectrum the spectrometer was zeroed to account for any stray light. The background spectrum was taken using a sample from the same PBS batch utilized in the preparation of the RBCs suspensions. Twenty microlitres of prepared RBC suspension was added to 2 ml of PBS in a quartz cuvette and inverted to ensure homogeneous distribution of RBCs in the cuvette. For each culture sample, 5–8 replicate spectral measurements were taken. The spectral measurements were taken at 10–11, 15–17, and 22 (for MFQ treated and control cultures) hrs post treatment.

### Spectral interpretation analysis

**Figure 1:**
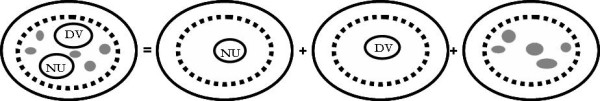
**Schematics of the *P. falciparum *infected RBC model.** IRBC was approximated as a weighted sum of three structural groups each having three concentric layers. The outer layer was IRBC cytosol, the middle layer (dashed line) was the parasite’s cytoplasm and the core was either digestive vacuole (DV), nucleus (NU), or organelles (ORG).

The structure of the *spectral interpretation model for IRBCs was described in detail in Serebrennikova et* al. [25]. In brief, the model included a three-layer Mie geometry (i.e., a sphere having three concentric layers) to account for changes in the refractive indices of the IRBC cytosol and the parasite’s cytoplasm and organelles. The model incorporated an ellipsoidal approximation to Mie theory to account for the effects of the RBC orientation and non-spherical shape. It has been recently demonstrated that this approximation can accurately predict the spectral features of oriented composite ellipsoids [27]. The ellipsoidal approximation to the RBC shape is quantitatively suitable for the prediction of light attenuation by RBCs in the forward direction [28]. According to the model the measured optical density was theoretically predicted as a weighted sum of the extinctions of orientation populations:

(1) $$\tau {\left(\lambda \right)}_{\text{calc}}=N_{p}L\sum \omega_{i}\left(A_{i}/4\right)Q_{\text{ext},i}$$

where *τ(λ)*_*calc*_ is the predicted total optical density at given wavelength *λ*, N_p_ is the total number density of cells, L is the pathlength, *ω*_*1*_ is the weight fraction of the *i*^*th*^ orientation, *A*_*i*_ and *Q*_*ext,i*_ are the projected area and extinction, respectively, of the cells at the *i*^*th*^ orientation. The extinction *Q*_*ext*_ of each IRBC orientation was, in turn, computed as weighted sum of the extinctions of three structural groups (Figure 1):

(2) $$Q_{\text{ext},i}=\omega_{\mathrm{DV}}Q_{\text{ext},\mathrm{DV}}+\omega_{\mathrm{NU}}Q_{\text{ext},\mathrm{NU}}+\omega_{\text{ORG}}Q_{\text{ext},\text{ORG}}$$

Each structural group was modelled as a three-layer structure such that the outer layer was the IRBC cytosol, the intermediate layer was the parasite’s cytoplasm, and the core was a parasite’s structural element. Three structural elements were suggested by the available information on the morphological structure of *P. falciparum* cells [15]. Those were digestive vacuole (DV) containing haemozoin, nucleus (NU) containing nucleic acid that provides distinct spectral contribution, and the third structural element was the population of other protozoan organelles. This “average” organelle (ORG) was modelled using the average refractive index of non-chromophoric molecules of biological cells [29]. Validated approximation of additivity of the spectral contributions from the structural groups [25,29] was used. The extinction of each structural group was a function of the size parameters and refractive indices of the layers. The size parameter of each layer (χ) was defined as:

(3) $$\chi =2\pi n_{0}D_{\text{MIE}}/\lambda$$

where *n*_*0*_ is the refractive index of the medium and *D*_*MIE*_ is the diameter. In the case of the outer layer (i.e., RBC cytosol) *D*_*MIE*_ denotes the equivalent sphere diameter of the ellipsoid computed according to [30] as:

(4) $$D_{\text{MIE}}=a/g$$

(5) $$g={\left({\text{cos}}^{2}\psi +\left({\text{cos}}^{2}\xi +r^{2}{\text{sin}}^{2}\xi \right){\text{sin}}^{2}\psi \right)}^{1/2}$$

where *r* = *a/c* is the ratio of the symmetry axis *a* to minor axis *c* of the ellipsoid and *ψ* and *ξ* are the rotation angles of the ellipsoid’s *x-z* and *y-z* planes with respect to the direction of the incident beam. The real *n* and imaginary *k* parts of the complex refractive index of the *l*^th^ layer were computed as weighted sums of the refractive indices of its compositional constituents:

(6) $$k_{l}=\sum \upsilon_{\mathrm{lj}}k_{j}$$

(7) $$n_{l}=\sum \upsilon_{\mathrm{lj}}n_{j}$$

where *υ*_*ij*_ is the weight fraction of the *j*^th^ compositional constituent in the *l*^th^ layer (Table 1). The outer layer was modelled to be composed of haemoglobin and water. The intermediate layer (i.e. parasite cytoplasm) was modelled to be composed of water and non-absorbing macromolecules [29]. The core elements were modelled as following: nucleus was composed of nucleic acid, non-absorbing macromolecules, and water; digestive vacuole was composed of water and haemozoin [25]; and the “average” organelle was composed of water and non-absorbing macromolecules. The refractive index of nucleic acid was constructed from those of the purine and pyrimidine bases [29] assuming G + C fraction of 0.2 for *P. falciparum*[31].

**Table 1:** The composition of the structural groups of the IRBC interpretation model

**Structural group (shell/core):**	**Composition of the shell**	**Composition of the shell**	**Composition of the core**
IRBC body/			Haemozoin
Parasite body/			Haemoglobin
Digestive vacuole	Haemoglobin	Proteins	Water
IRBC body/			Proteins
Parasite body/	Water	RNA	DNA + RNA + nucleotides
Nucleus		Water	Water
IRBC body/			Proteins
			Water
Parasite body/			
Organelles			

The interpretation procedure consisted of the prediction of the measured spectra as functions of the model parameters and subsequent comparison of the predicted and measured spectra using an iterative least squares minimization procedure. The residual sum of squares (RSSQ) was calculated as:

(8) $$\text{RSSQ}=\left(1/M\right){\sum \left(\tau {\left(\lambda \right)}_{\text{meas},m}-\tau {\left(\lambda \right)}_{\text{calc},m}\right)}^{2}$$

where M = 651 and corresponds to the 250–900 nm wavelength range and 1 nm wavelength resolution. Model parameters were iterated until convergence (i.e., relative changes in RSSQ were less than 10^-5^) using a Nelder-Meade downhill simplex optimization algorithm and variable transformation techniques [14,25].

The outputs of the interpretation analysis were used to reconstruct the measured spectra of IRBCs. In order to eliminate the orientation effect [25] and, therefore, to directly compare the effects of the structural and compositional differences, the IRBC spectra were reconstructed with the same orientation parameters. The interpretation of the measured UV-visible-NIR spectra of *P. falciparum* cells extracted from IRBCs was conducted using two-layer Mie theory as described in detail in [14].

## Results and discussion

### Untreated parasites

**Figure 2:**
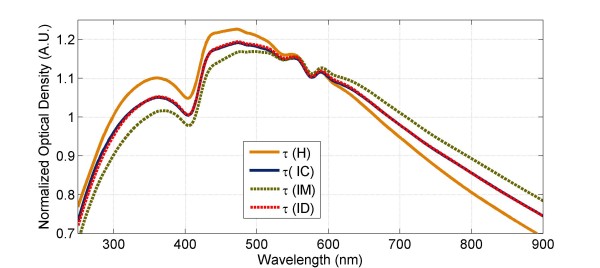
Reconstructed spectra of non-infected healthy RBCs (H), control IRBCs (IC), MFQ treated IRBCs (IM), and DHA treated IRBCs (ID) at 10–11 hrs post treatment.

**Figure 3:**
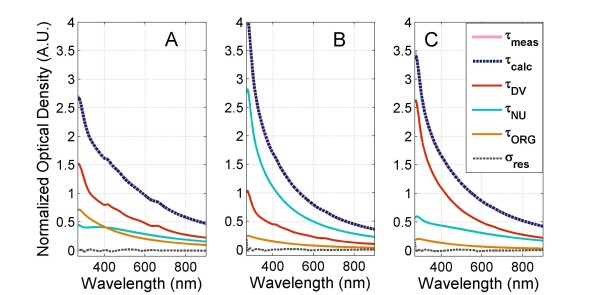
**Interpretation of the spectra of *P. falciparum *parasites extracted from IRBCs at 15–17 hrs post treatment: (A) controls, (B) DHA treated, (C) MFQ treated.** The measured spectra (τ_meas_), corresponding reconstructed spectra (τ_calc_), and predicted spectral contribution from the nucleus (τ_NU_), organelles (τ_ORG_), and digestive vacuole (τ_DV_) structural components are compared in each subplot. σ_res_ denotes the difference between the measured and predicted spectra.

The average reconstructed spectra of non-infected healthy RBCs (H), control IRBCs (IC), MFQ treated IRBCs (IM), and DHA treated IRBCs (ID) at 10–11 hrs post treatment are contrasted in Figure 2. Significant differences in the spectral features could be noted. Comparison of non-infected RBCs and IRBC controls revealed spectral changes that were due to the structural and compositional alterations to IRBCs caused by intraerythrocytic development of parasites. The interpretation models allowed the calculation of parameters relating to the morphology and haemoglobin composition of IRBCs as well as the size, structure, nucleotide and haemozoin composition of parasites from the measured spectra of IRBCs and extracted parasites. These structural and compositional parameters of the parasites in control cultures are given in Table 2. Figure 3 compares their measured and reconstructed spectra as well as the spectral contributions of the modelled structural groups. The structural and compositional parameters of IRBC control cultures are given in Table 3.

**Table 2:** **Structural and compositional parameters of *P. falciparum *obtained through interpretation of the measured UV-visible-NIR spectra of control IRBCs and parasites extracted from the host IRBCs**

**Paramer**	**10-11 hrs**	**15-17 hrs**	**22 hrs**
Average cell volume (fl)	16.0 ± 0.7	20.7 ± 0.4	22.4 ± 1.9
DV volume (fl)	3.9 ± 0.2	5.4 ± 0.2	6.0 ± 0.2
NU volume (fl)	2.8 ± 0.4	2.7 ± 0.5	2.8 ± 0.3
HZ (pg/cell)	0.15 ± 0.03	0.28 ± 0.04	0.27 ± 0.01
Nucleotides (fg/cell)	45 ± 12	141 ± 30	120 ± 21

**Table 3:** Structural and compositional parameters of non-infected healthy RBCs and control IRBCs obtained through interpretation of measured UV-visible-NIR spectra

**Parameter**	**Non-infected RBCs**		**IRBCs**	
		**10-11 hrs**	**15-17 hrs**	**22 hrs**
RBC volume (μm^3^)	83.1 ± 0.7	85.6 ± 1.0	87.8 ± 1.4	99.6 ± 1.8
RBC length (μm)	8.45 ± 0.03	8.45 ± 0.03	8.26 ± 0.05	7.95 ± 0.06
RBC width (μm)	2.22 ± 0.02	2.29 ± 0.02	2.47 ± 0.06	2.98 ± 0.08
MCHC (g ml^-1^)	30.4 ± 0.5	32.0 ± 0.0	28.3 ± 0.6	24.0 ± 0.1
MCH (pg cell^-1^)	25.5 ± 0.6	21.6 ± 1.0	20.0 ± 0.7	18.2 ± 0.5
MCH_I_ (pg cell^-1^)	-	25.7 ± 0.6	25.1 ± 0.6	25.7 ± 0.7

*MCHC* mean corpuscular haemoglobin concentration,

*MCH* mean corpuscular haemoglobin amount per cell,

*MCHC*_I_ estimated initial MCH of IRBCs.

Comparison between the parameters of control parasites obtained at 10–11 and 15–22 hrs revealed progressive changes in the sizes of the cells and organelles as well as haemozoin and nucleotide content of the cells indicating growth and metabolic activity. For instance, the estimated cell volume for *P. falciparum* increased from 16 μm^3^ at 10–11 hrs to 20–22 μm^3^ at 15–22 hrs. These values showed a transition from the ring to trophozoite stages paralleling other reports of cell volumes (10 ± 5 μm^3^ and 30 ± 10 μm^3^)for the ring and trophozoite stages, respectively [15,17]. The digestive vacuole volume has been reported to range from 4 μm^3^ at the early trophozoite stage [15,24] to 9–11 μm^3^ at later developmental stages [20]. The DV volumes estimated in this study were in good agreement with published data and also suggested early trophozoite stage at 10–11 hrs and more advanced mid-trophozoite stage at 15–22 hrs for the controls. Published micrographs of *P. falciparum* suggest that the nucleus occupies about 10-25% of the cell volume [15] and are in agreement with nucleus volumes obtained in this study (2.2-2.8 μm^3^) (Table 2). The amount of total nucleotide in control parasites (Table 2) was in agreement with our previous estimates made for *P. falciparum* trophozoite stage [14]. The amounts of haemozoin were 0.15 pg/cell and 0.27-0.3 pg/cell at 10–11 hrs and 15–22 hrs, respectively, for the controls. These values also corresponded well to the early and and mid-trophozoite stages of *P. falciparum*[23,24].

Even though control IRBCs retained the same volume as non-infected RBCs at 10–11 hrs, the cell aspect ratio (i.e., the ratio of the width to length) increased indicating changes in the cell shape (Table 3). The haemoglobin content of control IRBCs was also reduced due to its uptake by the parasites. The volume and aspect ratio of control IRBCs continued to increase whereas haemoglobin content continued to decrease from the 10–11 to 15–17 and to 22 hrs measurements in response to the intraerythrocytic growth of *P. falciparum* (Table 3). The changes in the aspect ratio indicated swelling of IRBCs; IRBC swelling has been previously demonstrated to occur in response to the parasite growth through trophozoite stage [20,32,33].

Haemoglobin losses from the IRBC cytosol to the parasite were 15% at 10–11 hrs, 20% at 15–17 hrs, and up to 30% at 22 hrs. These values were in agreement with those from other studies utilizing other approaches such as refractive index mapping and colorimetric iron determination. These reports indicate 5-10% and 40-60% haemoglobin depletion from the host at the ring and trophozoite stages, respectively [17,23]. Using the estimated values of the IRBCs’ MCH (Table 3) and haemozoin amount accumulated by parasites (Table 2), total iron of IRBCs was computed and expressed it as MCH_I_ (Table 3). These values were in good agreement with the MCH values of non-infected RBCs indicating no net loss of iron from IRBCs. No haemoglobin leakage from IRBCs despite parasite activity has been previously suggested [23].

### Mefloquine studies

**Figure 4:**
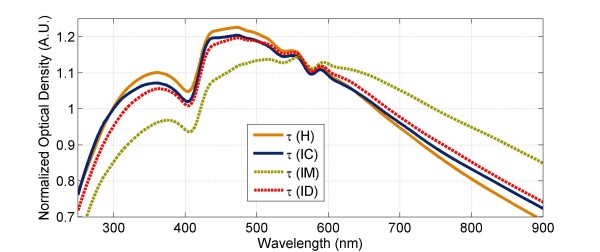
Reconstructed spectra of non-infected healthy RBCs (H), control IRBCs (IC), MFQ treated IRBCs (IM), and DHA treated IRBCs (ID) at 15–17 hrs post-reatment.

The sensitivity to the drug treatment at 10–11 hrs could be appreciated when the spectra of MFQ and DHA treated IRBCs are compared to those of the controls (Figure 4). The spectral changes became even more pronounced at the subsequent 15–17 hrs measurements (Figure 4). The structural and compositional parameters of MFQ treated parasites obtained with the interpretation model are summarized in Table 4. The structural and compositional parameters of IRBCs are given in Table 5. Comparison between the estimated parameters of control (Table 2) and MFQ treated (Table 4) parasites reveals that both treated and control parasites synchronously grew in size and no significant differences in the cell volumes between the two can be noted at any measurement time point. However, the DV volume values of MFQ treated parasites, 6.7-8.5 μm^3^ (Table 4), were nearly two-fold larger than those of controls, 3.9-6.0 μm^3^ (Table 2). The swelling of the parasite’s DV has been reported as one of the MFQ effects on the parasite morphology [34]. Remarkably, cellular haemozoin levels for MFQ treated parasites, 0.03-0.04 pg (Table 4), were substantially reduced compared to that of controls, 0.15-0.28 pg (Table 2). This difference became more pronounced in the 15–22 hrs measuments. Although nucleus volume appeared to be unaffected by MFQ treatment, the estimated amount of total nucleotides per cell for MFQ treated parasites was only about a half of that for the controls (Tables 2 and 4).

**Table 4:** **Structural and compositional parameters of *P. falciparum *obtained through interpretation of the measured UV-visible-NIR spectra of MFQ treated IRBCs and parasites extracted from IRBCs**

**Paramer**	**10-11 hrs**	**15-17 hrs**	**22 hrs**
Average cell volume (fl)	15.6 ± 0.3	21.7 ± 0.9	20.4 ± 1.6
DV volume (fl)	6.7 ± 0.3	7.6 ± 0.3	8.5 ± 0.9
NU volume (fl)	2.1 ± 0.1	2.2 ± 0.4	3.4 ± 0.2
HZ (pg/cell)	0.03 ± 0.01	0.03 ± 0.01	0.04 ± 0.01
Nucleotides (fg/cell)	26 ± 13	64 ± 3	84 ± 11

**Table 5:** Structural and compositional parameters of IRBCs obtained through interpretation of the measured UV-visible-NIR spectra of MFQ treated cultures

**Parameter**	**10-11 hrs**	**15-17 hrs**	**22 hrs**
RBC volume (μm^3^)	85.0 ± 0.9	85.1 ± 1.2	97.3 ± 1.0
RBC length (μm)	8.41 ± 0.07	8.31 ± 0.02	8.15 ± 0.01
RBC width (μm)	2.30 ± 0.4	2.36 ± 0.04	2.88 ± 0.04
MCHC (g ml^-1^)	35.0 ± 1.0	38.0 ± 0.1	32.0 ± 0.1
MCH (pg cell^-1^)	24.4 ± 0.4	24.0 ± 0.5	24.4 ± 0.5
MCH_I_ (pg cell^-1^)	25.2 ± 0.2	24.7 ± 0.4	25.5 ± 0.3

Although the cell size of MFQ treated parasites increased between the 10–11 and 15–22 hrs measurements, lack of continuous haemozoin production and lower total nucleotide levels suggest reduced metabolic activity compared to that of the controls. The differences in these values between control and MFQ treated parasites are already apparent at 10–11 hrs measurements (Tables 2 and 4). A comparison between the measured spectra of control and MFQ treated parasites also reveals the expected absence of a haemozoin peak at 650 nm [15] (Figure 3). The greater contribution of the DV spectral features to the reconstructed spectrum of MFQ treated parasites was also apparent in Figure 3.

Since MFQ treated parasites stopped producing haemozoin, the host IRBCs were not as depleted of their haemoglobin content as the IRBC controls. For MFQ treated IRBCs haemoglobin depletion was only 3% at all measurement time points. Even though MFQ treated parasites did not show haemozoin production, the size of MFQ treated IRBCs continued to increase with parasite growth as that of control IRBCs. Yet, the volume gained by MFQ treated IRBCs, i.e. the difference between the IRBC volume and non-infected RBC volume, was less than the parasite volume at any given time point. For instance, at 10–11 hrs the volume increase was 2 μm^3^ whereas the parasite volume was 16 μm^3^ and at 22 hrs the volume increase was 14 μm^3^ whereas the parasite volume was 20 μm^3^. In non-treated cultures, the parasites would have removed the excessive haemoglobin to reduce the IRBC volume gain and, therefore, to support the IRBC osmotic balance and integrity and to prevent the IRBC rupture. The results from MFQ treatment suggested that the integrity of MFQ treated IRBCs was still maintained by the parasites. They could have done it, for instance, by removing the excessive water from the host IRBC cytosol. This suggestion is supported by the higher water to haemoglobin removal ratio calculated for treated IRBCs (~4) compared to that for non-treated IRBCs (~2). The spectra of MFQ treated IRBCs differred dramatically from those of control IRBCs at all measurement time points (Figures 2 and 3). The dominant cause of this distinction was the greater refractive index values of MFQ treated IRBC which have a greater haemoglobin content compared to that of non-treated IRBCs.

### Dihydroartemisinin studies

It has been reported that oxidative stress caused by DHA actually kills malaria parasites inside IRBCs [35]. Here we examine what it meant in terms of the changes in the structure and composition of parasites and the effects of those changes on the spectral features of DHA treated IRBCs. The structural and compositional parameters of DHA treated parasites obtained through interpretation of the spectra measured at 10–11 and 15–17 hrs are summarized in Table 6. The structural and compositional parameters of DHA treated IRBCs are given in Table 7. All parameters estimated for DHA treated parasites were already different from those of the controls at 10–11 hrs post treatment. The cell size and organelle volumes were about 30% lower for DHA treated parasites than those for the controls. Similarly to MFQ treated parasites, DHA treated parasites did not accumulate haemozoin. The total nucleotide values for DHA treated parasites were considerably lower than those for the controls and MFQ treated parasites. The differences were already dramatic at 10–11 hrs and increased at the next measurement time point. All parameter values estimated for DHA treated parasites remained fairly unchanged between the 10–11 and 15–17 hrs measurements. This consistency suggests no parasite growth between measurements; therefore, DHA treated parasites were not alive by the 10–11 hrs measurement and the structural and compositional characteristics captured reflected those of dead cells. The spectral features of DHA treated parasites were distinguished from those of the controls and MFQ treated parasites by the steeper slope indicative of the smaller cell size and the reduced peak at 650 nm indicative of the smaller haemozoin content (Figure 3).

**Table 6:** **Structural and compositional parameters of *P. falciparum *obtained through interpretation of the measured UV-visible-NIR spectra of DHA treated IRBCs and parasites extracted from IRBCs**

**Paramer**	**10-11 hrs**	**15-17 hrs**
Average cell volume (fl)	10.5 ± 1.3	10.2 ± 2.1
DV volume (fl)	2.9 ± 0.3	3.0 ± 0.3
NU volume (fl)	1.8 ± 0.3	2.0 ± 0.8
HZ (pg/cell)	0.05 ± 0.03	0.04 ± 0.02
Nucleotides in NU (fg/cell)	15 ± 13	14 ± 2

**Table 7:** Structural and compositional parameters of IRBCs obtained through interpretation of the measured UV-visible-NIR spectra of DHA treated cultures

**Parmeter**	**10-11 hrs**	**15-17 hrs**
RBC volume (μm^3^)	82.9 ± 0.7	89.1 ± 2.2
RBC length (μm)	8.37 ± 0.02	8.26 ± 0.07
RBC width (μm)	2.27 ± 0.02	2.41 ± 0.09
MCHC (g ml^-1^)	32.6 ± 0.55	30.4 ± 0.9
MCH (pg cell^-1^)	23.6 ± 0.5	24.1 ± 0.4
MCH_I_ (pg cell^-1^)	25.0 ± 0.5	25.3 ± 0.4

Similarly to MFQ treatment, haemoglobin depletion of DHA treated IRBCs was significantly reduced compared to that of IRBC controls. DHA treated IRBCs were depleted by only 5% in haemoglobin content at 10–11 hrs and remained such at 15–17 hrs. The spectral interpretation analysis showed that DHA treated IRBCs increased in volume from the 10–11 hrs to 15–17 hrs measurements (Table 7). Since the parasite size did not increase between the measurements and haemoglobin content remained the same, this volume gain could be attributed to the influx of water into DHA treated IRBCs. This allowed DHA treated IRBCs to regain their initial MCHC value that was disturbed by the parasite invasion (Table 7). Such measure resulted in the reduced refractive index values of DHA treated IRBCs compared to MFQ treated IRBCs. The refractive index of the cells has a pronounced influence on the spectral features. The refractive indices of control IRBCs and DHA treated IRBCs were practically the same due to similar MCHC values at 10–11 hrs measurement (Tables 3 and 7). In addition, given the close sizes of IRBCs and parasites between controls and DHA treated cultures, the spectral features of control and DHA treated IRBCs overlapped above 400 nm at 10–11 hrs post treatment (Figure 2). However, subsequent changes in the refractive index of control IRBCs attributable to the parasite growth and haemoglobin depletion led to greater discrimination between the spectral features of control and DHA treated IRBCs at 15–17 hrs measurement (Figure 4).

## Conclusion

A comprehensive spectral interpretation model was developed in order to extract information on the structure and composition of the intraerythrocytic parasite and its host from spectroscopic measurements. Quantitative analysis of the UV-visible spectra of IRBCs parasitized with the most lethal malaria parasite *P. falciparum* allowed insights into the parasite and host interactions. These results appeared to be robust when compared to the reported measured values and estimates made with other methods.

Notable changes occur in the IRBC spectral features due to reshaping of the infected cells, growth of intracellular parasites, and depletion of haemoglobin from the host IRBC cytosol. The spectral interpretation analysis indicated a mass balance between the total iron in the IRBCs (haemoglobin in cytosol plus haemozoin in parasites) and that in healthy RBCs (haemoglobin) indicating no net loss of iron from IRBCs despite a parasite-induced increase in the permeability of the IRBC membrane. IRBCs increased in volume and became more spherical with parasite’s progression from the ring stage into trophozoite stage. Because of the parasite’s effort to maintain the osmotic balance and integrity of the host IRBC, the IRBC volume was less than a sum of those of a healthy RBC and a parasite at trophozoite stage. This effort included excessive uptake and break up of haemoglobin [36] followed by the conversion of haem into haemozoin pigment and removal of free amino acids and other protein pieces [18,21].

Since spectroscopic measurements have high sensitivity to the size and composition of particles and cells, the action of anti-malarial drugs on the structure and composition of the parasites and IRBCs led to distinguishing spectral features. Our results demonstrated that the effects of MFQ and DHA could readily be appreciated at 10–11 hrs after drug administration. Specifically, MFQ blocked haemozoin formation and led to the swelling of the parasite’s digestive vacuole. Since the host IRBC was minimally depleted in haemoglobin, it increased in volume and changed shape with parasite’s growth. On the other hand, as DHA terminated the parasite’s metabolic activity, and it reduced the sizes of the parasite’s cell body and organelles. The cell and organelle volumes of DHA treated parasites were about 60-70% of those of non-treated parasites grown in parallel as controls. Haemoglobin depletion from the host cytosol and haemozoin accumulation discontinued when DHA treatment became effective. These findings suggested that a spectral interpretation analysis could have significant potential for drug effect evaluation of parasite isolates or for therapeutic monitoring of malaria patients.

## Competing interests

The authors declare that they have no competing interests.

## Authors’ contributions

YS performed the spectroscopic measurements, conducted data analysis, and drafted the manuscript. JP carried out parasite cultivation and treatment. JP and WM conceived and designed the culture experiments. LGR, DH, and JS contributed to the data interpretation and manuscript preparation. All authors had full access to all data in the study, read and approved the manuscript.
